# A Novel Hybrid Self-Adaptive Bat Algorithm

**DOI:** 10.1155/2014/709738

**Published:** 2014-04-09

**Authors:** Iztok Fister, Simon Fong, Janez Brest, Iztok Fister

**Affiliations:** ^1^Faculty of Electrical Engineering and Computer Science, University of Maribor, Smetanova 17, 2000 Maribor, Slovenia; ^2^Department of Computer and Information Science, University of Macau, Avenue Padre Tomas Pereira, Taipa, Macau

## Abstract

Nature-inspired algorithms attract many researchers worldwide for solving the hardest optimization problems. One of the newest members of this extensive family is the bat algorithm. To date, many variants of this algorithm have emerged for solving continuous as well as combinatorial problems. One of the more promising variants, a self-adaptive bat algorithm, has recently been proposed that enables a self-adaptation of its control parameters. In this paper, we have hybridized this algorithm using different DE strategies and applied these as a local search heuristics for improving the current best solution directing the swarm of a solution towards the better regions within a search space. The results of exhaustive experiments were promising and have encouraged us to invest more efforts into developing in this direction.

## 1. Introduction

Optimization has become more and more important, especially during these times of recession. It has been established throughout practically all spheres of human activities, for example, finances, industries, sport, pharmacy, and so forth. In each of these spheres, a goal of the optimization is to find the optimal input parameters according to known outcomes and a known model. This model represents a problem to be solved and transforms input parameters to output outcomes. Essentially, the optimal input parameters must be properly evaluated in order to determine the quality of a solution. Indeed, an objective function is used that mathematically describes this quality. In the praxis, the value of the objective function can be either minimized or maximized. For example, when buying a car either the minimum cost min⁡(*f*(*x*)) or maximum comfort max⁡(*f*(*x*)) is interesting objective for a potential buyer, where *f*(*x*) denotes the objective function. Using the equation min⁡(*f*(*x*)) = max⁡(−*f*(*x*)), the maximization problem can be transformed into a minimization one, and vice versa. The function expressed in this way is also named as fitness function in evolutionary computation community. In place of the objective function, the minimization of the fitness function is assumed in this paper.

Most of the problems arising in practice today are NP-hard. This means that the time complexity of an exhaustive search algorithm running on a digital computer which checks all solutions within a search space increases exponentially by increasing the instance size as determined by the number of input parameters. In some situations, when the number of input parameters increases to a certain limit, it can be expected that a user never obtains the results from the optimization. As a result, algorithms solving the NP-problems approximately have arisen in the past. Although these algorithms do not find the exact optimal solution, in general, their solutions are good enough in practice. For instance, the well-known algorithms for approximately solving the hardest problems today are

- artificial bee colony (ABC) [1],
- bat algorithm (BA) [2],
- cuckoo search (CS) [3],
- differential evolution (DE) [4],
- firefly algorithm (FA) [5, 6],
- particle swarm optimization (PSO) [7],
- many more [8].

The developers of the above-mentioned algorithms were typically inspired by nature in a development phase. Namely, each nature-inspired algorithm mimics the natural behaviors of specific biological, chemical, or physical systems in order to solve particular problems using a digital computer. Most of the inspiration from nature emanates from biology. The greatest lasting impression on the developers has been left by Darwin's evolution [9]. Darwin observed that nature is not static and therefore the fitter individuals have more chances of surviving within the changing environments. Holland in [10] connected this adaptive behavior of the natural systems to artificial systems that simulate their behavior by solving the particular problems (also optimization problems) on the digital computers.

Optimization algorithms are controlled by algorithm parameters that can be changed deterministically, adaptively, and self-adaptively [11]. Deterministic parameters are altered by using some deterministic rules (e.g., Rechenberg's 1/5 success rule [12]). In contrast, adaptively controlled parameters are subject to feedback from the search process that serves as an input to the mechanism used which determines the direction and magnitude of the change [11]. Finally, the self-adaptively controlled parameters are encoded into a representation of the solution and undergo the actions of variation operators [13].

This paper focuses on the adaptation and hybridization of a swarm intelligence algorithm. Swarm intelligence (SI) belongs to an artificial intelligence discipline (AI) which first became popular over the last decade and still is [14]. It is inspired by the collective behavior of social swarms of ants, termites, bees, and worms, flock of birds, and schools of fish [15]. Although these swarms consist of relatively unsophisticated individuals, they exhibit coordinated behavior that directs the swarms towards their desired goals. This usually results in the self-organizing behavior of the whole system, and collective intelligence or swarm intelligence is in essence the self-organization of such multiagent systems, based on simple interaction rules.

The bat algorithm (BA) is one of the younger members of this family which was developed by Yang [16]. It is inspired by the microbats that use an echolocation for orientation and prey seeking. The original bat algorithm employs two strategy parameters: the pulse rate and the loudness. The former regulates an improvement of the best solution, while the latter influences an acceptance of the best solution. Both mentioned parameters are fixed during the execution of the original bat algorithm. In the self-adaptive bat algorithm (SABA) developed by Fister et al. [17] these parameters are self-adaptive. The aim of this self-adaptation is twofold. On the one hand, it is very difficult to guess the valid value of the parameter at the beginning of the algorithm. On the other hand, this value depends on the phase in which the search process is. This means that the parameter setting at the beginning of the search process can be changed when this process reaches maturity. In this paper, a further step forward has been taken.

Hybridization with local search heuristics has now been applied in order to further improve the results of the SABA algorithm. Domain-specific knowledge can be incorporated using the local search. Although the local search is as yet an ingredient of the original bat algorithm it can be replaced by different DE strategies [18]. Indeed, the further improvement of the current best solution is expected which would direct the swarm intelligence search towards the more promising areas of the search space. As a result, the hybrid self-adaptive bat algorithm (HSABA) that was applied to a benchmark suite consisting of ten well-known functions from the literature was developed. The results of HSABA obtained during extensive experiments showed that the HSABA improved the results of both the original BA and the SABA. Moreover, the results were also comparable with the other well-known algorithms, like firefly (FA) [5], differential evolution (DE) [19], and artificial bee colony (ABC) [20].

The structure of this paper is as follows.  Section 2 presents an evolution of the bat algorithms from the original BA via the self-adaptive SABA to the hybrid self-adaptive HSABA algorithm.  Section 3 describes the experimental work, while Section 4 illustrates the obtained results in detail. The paper concludes by summarizing the performed work and outlines directions for the further development.

## 2. Evolution of Bat Algorithms

The results of experiments regarding the original bat algorithm showed that this algorithm is efficient especially when optimizing the problems of lower dimensions. In line with this, Eiben and Smith in [11] asserted that 2-dimensional functions are not suitable for solving with the population-based stochastic algorithms (like evolutionary algorithms and swarm intelligence), because these can be solved optimally using traditional methods. On the other hand, these kinds of algorithms could play a role as general problem solvers, because they share the same performances when averaged over all the discrete problems. This fact is the essence of the so-called No-Free Lunch (NFL) theorem [21]. In order for this theorem to prevail, there are almost two typical mechanisms for improving the performance of the population-based algorithms as follows:

- self-adaptation of control parameters,
- hybridization.

The former enables the control parameters to be changed during the search process in order to better suit the exploration and exploitation components of this search process [22], while the latter incorporates the problem-specific knowledge within it.

In the rest of this paper, firstly the original BA algorithm is presented in detail, followed by describing the application of a self-adaptive mechanism within the original BA algorithm which leads to the emergence of a self-adaptive BA algorithm SABA. Finally, a hybridization of the SABA algorithm is broadly discussed which obtains a hybridized SABA algorithm named the HSABA.

### 2.1. The Original Bat Algorithm

The original BA is a population-based algorithm, where each particle within the bat population represents the candidate solution. The candidate solutions are represented as vectors **x**_**i**_ = (*x*_*i*1_,…, *x*_*iD*_)^*T*^ for *i* = 1 ⋯ *Np* with real-valued elements *x*_*ij*_, where each element is drawn from interval *x*_*ij*_ ∈ [*x*_*lb*_ ⋯ *x*_*ub*_]. Thus, *x*_*lb*_ and *x*_*ub*_ denote the corresponding lower and upper bounds, and *Np* determines a population size.

This algorithm consists of the following main components:

- an initialization,
- a variation operation,
- a local search,
- an evaluation of a solution,
- a replacement.

In the* initialization*, the algorithm parameters are initialized, then, the initial population is generated randomly, next, this population is evaluated, and finally, the best solution in this initial population is determined. The* variation operator* moves the virtual bats in the search space according to the physical rules of bat echolocation. In the* local search*, the current best solution is improved by the random walk direct exploitation heuristics (RWDE) [23]. The quality of a solution is determined during the* evaluation of solution*. The* replacement* replaces the current solution with the newly generated solution regarding the some probability. This component is similar to the simulated annealing [24], where the new solution is accepted by the acceptance probability function which simulates the physical rules of annealing. The pseudocode of this algorithm is presented in Algorithm 1.

In Algorithm 1 the particular component of the BA algorithm is denoted either by function name when it comprises one line or by a comment between two curly brackets when the components are written within a structured statement and/or it comprises more lines. In line with this, the initialization comprises lines 1–3 in Algorithm 1, the variation operation line 6 (function* generate_new_solution*), the local search lines 7–9, the evaluation of the solution (function* evaluate_new_solution* in line 10), and the replacement lines 12–14. In addition, the current best solution is determined in each generation (function* find_best_solution* in line 15).

The variation operation which is implemented in function* generate_new_solution* moves the virtual bats towards the best current bat's position according to the following equations:

(1) Qi(t)=Qmin⁡+(Qmax⁡−Qmin⁡)N(0,1),vi(t+1)=vit+(xit−best)Qi(t),xi(t+1)=xi(t)+vi(t+1),

where *N*(0,1) is a random number drawn from a Gaussian distribution with zero mean and a standard deviation of one. A RWDE heuristics [23] implemented in the function* improve_the_best_solution* modifies the current best solution according to the following equation:

(2) $$\begin{array}{c} x^{\left(t\right)}=best+\epsilon A_{i}^{\left(t\right)}N\left(0,1\right), \end{array}$$

where *N*(0,1) denotes the random number drawn from a Gaussian distribution with zero mean and a standard deviation of one, *ϵ* being the scaling factor and *A*_*i*_^(*t*)^ the loudness.

A local search is launched with the probability of pulse rate *r*_*i*_. As already stated, the probability of accepting the new best solution in the component* save_the_best_solution_conditionaly* depends on loudness *A*_*i*_. Actually, the original BA algorithm is controlled by two algorithm parameters: the pulse rate *r*_*i*_ and the loudness *A*_*i*_. Typically, the rate of pulse emission *r*_*i*_ increases and the loudness *A*_*i*_ decreases when the population draws nearer to the local optimum. Both characteristics imitate natural bats, where the rate of pulse emission increases and the loudness decreases when a bat finds a prey. Mathematically, these characteristics are captured using the following equations:

(3) $$\begin{array}{c} A_{i}^{\left(t+1\right)}=\alpha A_{i}^{\left(t\right)},\;\;r_{i}^{\left(t\right)}=r_{i}^{\left(0\right)}\left[1-\exp\left(-\gamma \epsilon \right)\right], \end{array}$$

where *α* and *γ* are constants. Actually, the *α* parameter controls the convergence rate of the bat algorithm and therefore plays a similar role as the cooling factor in the simulated annealing algorithm.

In summary, the original BA algorithm is based on a PSO algorithm [7] which is hybridized with RWDE and simulated annealing heuristics. The former represents the local search that directs the bat search process towards improving the best solution, while the latter takes care of the population diversity. In other words, the local search can be connected with exploitation, while simulated annealing uses the exploration component of the bat search process. The exploitation is controlled by the parameter *r* and exploration by the parameter *A*. As a result, the BA algorithm is able to explicitly control the exploration and exploitation components within its search process.

### 2.2. The Self-Adaptive Bat Algorithm

Almost two advantages can be expected when the self-adaptation of control parameters is applied to a population-based algorithm [11]:

- control parameters need not be set before the algorithm's run,
- the control parameters are adapted during the run to the fitness landscape defined by the positions of the candidate solutions within the search space and their corresponding fitness values [25].

Normally, the self-adaptation is realized by encoding the control parameters into representation of candidate solutions and letting them undergo an operation of the variation operators. In this way, the self-adaptation of the BA control parameters (the loudness and the pulse rate) is considered.

This means that the existing representation of the candidate solutions consisting of problem variables **x**_*i*_^(*t*)^ = (*x*_*i*1_^(*t*)^,…, *x*_*iD*_^(*t*)^) is widened by the control parameters *A* ^(*t*+1)^ and *r* ^(*t*+1)^ to

(4) $$\begin{array}{c} x_{i}^{\left(t\right)}={\left(x_{i1}^{\left(t\right)},\ldots ,x_{iD}^{\left(t\right)},A^{\left(t\right)},r^{\left(t\right)}\right)}^{T},\;\text{for}\;i=1\cdots Np, \end{array}$$

where *Np* denotes the population size. The control parameters are modified according to the following equations:

(5) $$\begin{array}{c} A^{\left(t+1\right)}=\{\begin{array}{cc} A_{lb}^{\left(t\right)}+\text{ran}{\text{d}}_{0}\left(A_{ub}^{\left(t\right)}-A_{lb}^{\left(t\right)}\right) & \text{if}\;\;\text{ran}{\text{d}}_{1}<\tau_{1},\\ A^{\left(t\right)} & \text{otherwise}, \end{array} \end{array}$$

(6) $$\begin{array}{c} r^{\left(t+1\right)}=\{\begin{array}{cc} r_{lb}^{\left(t\right)}+\text{ran}{\text{d}}_{2}\left(r_{ub}^{\left(t\right)}-r_{lb}^{\left(t\right)}\right) & \text{if}\;\;\text{ran}{\text{d}}_{3}<\tau_{2},\\ r^{\left(t\right)} & \text{otherwise}. \end{array} \end{array}$$

Note that the parameters *τ*_0_ and *τ*_1_ denote the learning rates that were set, as *τ*_0_ = *τ*_1_ = 0.1, while rand_*i*_ for *i* = 1 ⋯ 4 designate the randomly generated value from interval [0,1].

The self-adapting part of the SABA algorithm is performed by the* generate_the_new_solution* function (line 6 in Algorithm 1). Note that the control parameters are modified in this function according to the learning rates *τ*_0_ and *τ*_1_. In the case *τ*_0_ = *τ*_1_ = 0.1, each 10th candidate solution is modified on average. The modified parameters influence the application of the local search and the probability of the replacement. The replacement function preserves the problem variables as well as the control parameters.

This self-adapting function was inspired by Brest et al. [4] who proposed the self-adaptive version of DE, better known as jDE. This self-adaptive algorithm improves the results of the original DE significantly by continuous optimization.

### 2.3. The Hybrid Self-Adaptive Bat Algorithm

The population-based algorithms, like evolutionary algorithms and swarm intelligence, can be seen as some kind of general problem solvers, because they are applicable for all classes of optimization problems. Thus, their results confirm the so-called No-Free Lunch (NFL) theorem by Wolpert and Macready [21]. According to this theorem any two optimization algorithms are equivalent when their performances are averaged across all possible problems.

In order to circumvent the NFL theorem by solving a specific problem, domain-specific knowledge must be incorporated within the algorithm for solving it. The domain-specific knowledge can be incorporated by the bat algorithm within each of its components, that is, an initialization, a variation operator, a local search, an evaluation function, and a replacement.

Although the SABA algorithm significantly outperformed the results of the original BA algorithm, it suffers from a lack of incorporated domain-specific knowledge of the problem to be solved. This paper focuses on hybridizing the SABA using a novel local search heuristics that better exploits the self-adaptation mechanism of this algorithm. The standard “rand/1/bin” DE strategy and three other DE strategies focusing on the improvement of the current best solution were used for this purpose, where the modification of the local search in a hybrid self-adaptive BA algorithm (HSABA) is illustrated in Algorithm 2.

The local search is launched according to a threshold determined by the self-adapted pulse rate *r*_*i*_ (line 1). This parameter is modified by each 10th virtual bat, on average. The local search is an implementation of the operators crossover and mutation borrowed from DE [19]. Firstly, the four virtual bats are selected randomly from the bat population (line 2) and the random position is chosen within the virtual bat (line 3). Then, the appropriate DE strategy modifies the trial solution (lines 4–9) as follows:

(7) $$\begin{array}{c} y_{n}=\{\begin{array}{cc} \text{DE}\_\text{Strategy}, & \begin{array}{c} \text{if}\;\;\text{rand}\left(0,1\right)\leq \text{CR}\vee n=D, \end{array}\\ x_{\text{in}}^{\left(t\right)} & \text{otherwise}, \end{array} \end{array}$$

where the DE_Strategy is launched according to the probability of crossover CR. The term *n* = *D* ensures that almost one modification is performed on the trial solution. The DE*_*Strategy function is presented in Algorithm 3.

Although more different DE strategies exist today, we have focused mainly on those that include the current best solution in the modification of the trial solution. These strategies are presented in Table 1. Note that the suitable DE strategy is selected using the global parameter strategy.

The strategies illustrated in the table that use the best solution in the modification operations, that is, “randToBest/1/bin,” “best/2/bin,” and “best/1/bin,” typically direct the virtual bats towards the current best solution. Thus, it is expected that the new best solution is found when the virtual bats move across the search space directed by the best solution. The “rand/1/bin” represents one of the most popular DE strategies today that introduces an additional randomness into a search process. Obviously, it was used also in this study.

## 3. Experiments

The goal of our experimental work was to show that the HSABA outperforms the results of the original BA as well as the self-adaptive SABA algorithms, on the one hand, and that these results were comparable with the results of other well-known algorithms, like firefly (FA) [5], differential evolution (DE) [19], and artificial bee colony (ABC) [20]. All the mentioned algorithms were applied to a function optimization that belongs to a class of combinatorial optimization problems.

Function optimization is formally defined as follows. Let us assume a fitness function *f*(**x**), where **x** = (*x*_1_,…, *x*_*D*_) denotes a vector of *D* design variables from a decision space *x*_*j*_ ∈ *S*. The values of the design variables are drawn from the interval *x*_*j*_ ∈ [*lb*_*j*_, *ub*_*j*_], where *lb*_*j*_ ∈ ℝ and *ub*_*j*_ ∈ ℝ are their corresponding lower and upper bounds, respectively. Then, the task of function optimization is to find the minimum of this objective function.

In the remainder of this paper, the benchmark function suite is presented, then the experimental setup is described, and finally, the configuration of the personal computer (PC) on which the tests were conducted is clarified in detail.

### 3.1. Benchmark Suite

The benchmark suite was composed of ten well-known functions selected from various publications. The reader is invited to check deeper details about test functions in the state-of-the-art reviews [26–28]. The definitions of the benchmark functions are summarized in Table 2 which consists of three columns denoting the function tag *f*, the* function name*, and the function* definition*.

Each function from the table is tagged with its sequence number from *f*_1_ to *f*_10_. Typically, the problem becomes heavier to solve when the dimensionality of the benchmark functions is increased. Therefore, benchmark functions of more dimensions needed to be optimized during the experimental work.

The properties of the benchmark functions can be seen in Table 3 which consists of five columns: the function tag *f*, the value of the optimal solution f* = *f*(x*), the optimal solution x*, the function* characteristics*, and* domain*. One of the more important characteristics of the functions is the number of local and global optima. According to this characteristic the functions are divided into either unimodal or multimodal. The former type of functions has only one global optimum, while the latter is able to have more local and global optima throughout the whole search space. Parameter* domain* limits the values of parameters to the interval between their lower and upper bounds. As a matter of fact, these bounds determine the size of the search space. In order to make the problems heavier to solve, the parameter domains were more widely selected in this paper than those prescribed in the standard publications.

### 3.2. Experimental Setup

The characteristics of the HSABA were observed during this experimental study. Then, the best parameter setting was determined. Finally, the results of the HSABA were compared with the results of the original BA and self-adaptive SABA algorithms. The results of the other well-known algorithms, like FA, DE, and ABC, were also added to this comparative study. Note that the best parameter settings for each particular algorithm were used in the tests, except BA algorithms, where the same setup was employed in order to make the comparison as fair as possible. All the parameter settings were found after extensive testing.

During the tests, the BA parameters were set as follows: the loudness *A*_0_ = 0.5, the pulse rate *r*_0_ = 0.5, minimum frequency *Q*_max⁡_ = 0.0, and maximum frequency *Q*_max⁡_ = 2.0. The same initial values for *r*_0_ and *A*_0_ were also applied by SABA and HSABA, while the frequency was captured from the same interval *Q* ∈ [0.0, 2.0] as by the original bat algorithm. Thus, the values for loudness are drawn from the interval *A* ^(*t*)^ ∈ [0.9,1.0] and the pulse rate from the interval *r* ^(*t*)^ ∈ [0.001,0.1]. The additional parameters controlling behavior of DE strategies were set as *F* = 0.01 and CR = 1.0. Both parameters had a great influence on the results of the HSABA algorithm, while this setting initiated the best performance by the self-adaptive SABA framework hybridized with DE strategies.

FA ran with the following set of parameters: *α* = 0.1, *β* = 0.2, and *γ* = 0.9, while DE was configured as follows: the amplification factor of the difference vector *F* = 0.5 and the crossover control parameter CR = 0.9. In the ABC algorithm, the onlooker bees represented 50% of the whole colony, while the other 50% of the colony was reserved for the employed bees. On the other hand, the scout bee was generated when its value had not improved in 100 generations. In other words, the parameter *limits* was set to the value 100.

Each algorithm in the tests was run with a population size of 100. Each algorithm was launched 25 times. The obtained results of these algorithms were aggregated according to their best, the worst, the mean, the standard deviation, and the median values reached during 25 runs.

### 3.3. PC Configuration

All runs were made on HP Compaq with the following configurations.

1. Processor: Intel Core i7-2600 3.4 (3.8) GHz.
2. RAM: 4 GB DDR3.
3. Operating system: Linux Mint 12.

Additionally, all the tested algorithms were implemented within the Eclipse Indigo CDT framework.

## 4. The Results

Our experimental work consisted of conducting the four experiments, in which the following characteristics of the HSABA were tested:

- an influence of using the different DE strategies,
- an influence of the number of evaluations,
- an influence of the dimensionality of problems,
- a comparative study.

In the first test, searching was performed for the most appropriate DE strategy incorporated within the HSABA algorithm. This strategy was then used in all the other experiments that followed. The aim of the second experiment was to show how the results of the HSABA converge according to the number of fitness function evaluations. Dependence of the results on the dimensionality of the functions to be optimized is presented in the third experiment. Finally, the results of the HSABA were compared with the original BA and self-adaptive SABA algorithms as well as the other well-known algorithms, like FA, DE, and ABC.

In the remainder of this paper, the results of the experiments are presented in detail.

### 4.1. Influences of Using the Different DE Strategies

**Figure 1:**
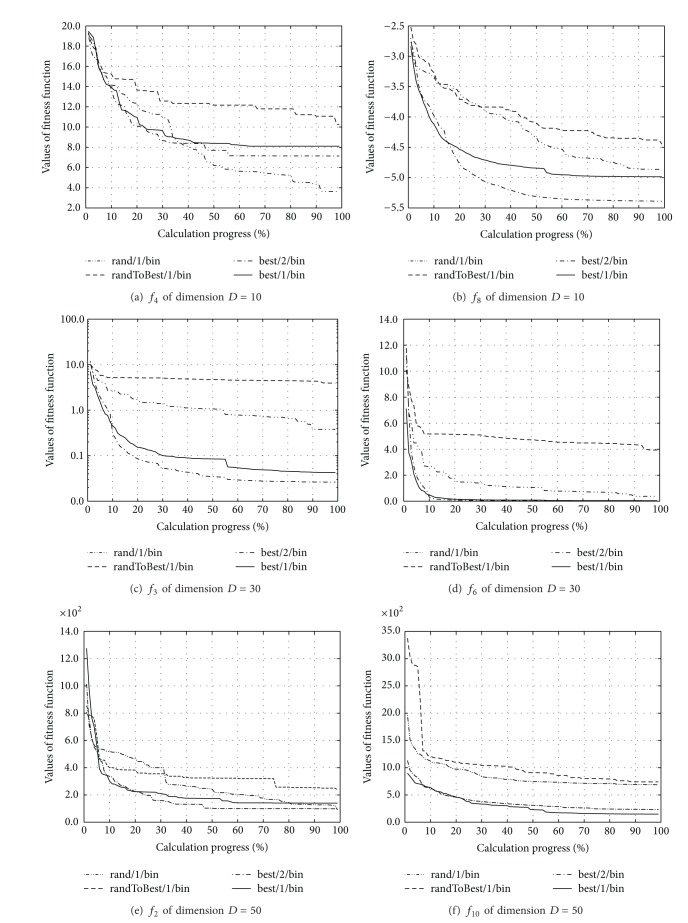
Impact of DE strategies on the results of optimization.

In this experiment, the qualities of four different DE strategies that were implemented within the HSABA algorithm were observed. The outcome of this experiment had an impact on the further tests, because all the tests that followed were performed with the most promising DE strategy found during the experiment. In line with this, each of the DE strategies, that is, “rand/1/bin,” “randToBest/1/bin,” “best/2/bin,” and “best/1/bin,” was tested by optimizing the benchmark function suite. The results of function optimization were averaged after 25 runs and are illustrated in Figure 1.

This figure is divided into six diagrams, two for each observed dimension of the function. Each diagram represents the progress of the optimization in percent on the *x*-axis, where 100% denotes the corresponding number of fitness function evaluations (e.g., 10,000 for 10-dimensional functions), while the value of the fitness function is placed alongside the *y*-axis.

In summary, the “best/2/bin” strategy achieved the best result when compared with the other three strategies, on average. Interestingly, the “rand/1/bin” strategy was the most successful when optimizing the function *f*_4_ of dimension *D* = 10. This behavior was in accordance with the characteristics of this function, because highly multimodal functions demand more randomness during the exploration of the search space.

### 4.2. Influence of the Number of Evaluations

A long-term lack of improvement regarding the best result during the run was one of the most reliable indicators of the stagnation. If the fitness value did not improve over a number of generations, this probably means that the search process got stuck within a local optimum. In order to detect this undesirable situation during the run of HSABA, the fitness values were tracked at three different phases of the search process, that is, at 1/25, at 1/5, and at the final fitness evaluation. Thus, HSABA runs with the “best/2/bin” strategy and parameters as presented in Section 3.2. The results of this test are collated in Table 4.

It can be seen from Table 4 that HSABA successfully progressed towards the global optimum according to all benchmark functions, except *f*_7_ which fell into a local optimum very quickly and did not find any way of further improving the result.

### 4.3. Influence of the Dimensionality of the Problem

The aim of this experiment is to discover how the quality of the results depends on the dimension of the problem, in other words, the dimensionality of the functions to be optimized. In line with this, three different dimensions of the benchmark functions *D* = 10, *D* = 30, and *D* = 50 were taken into account. The results of the tests according to five measures are presented in Table 5.

In this experiment, it was expected that the functions of the higher dimensions would be harder to optimize and therefore the obtained results would be worse. The results in Table 5 show that our assumption held for the average fitness values in general, except for function *f*_9_, where optimizing this function of dimension *D* = 10 returned the worst fitness value as by functions of dimensions *D* = 30 and *D* = 50.

### 4.4. Comparative Study

The true value of each algorithm is shown for only when we compared it with the other algorithms solving the same problems. Therefore, the HSABA was compared with algorithms such as BA, SABA, FA, DE, and ABC. All the algorithms were solved using the same benchmark functions as proposed in Section 3.1 and used the parameter setting as explained in Section 3.2. The results of this comparative study obtained by optimizing the functions of dimension *D* = 30 are illustrated in Table 6. The best results in these tables are presented as bold.

It can be seen from Table 6 that the HSABA outperformed the results of the other algorithms sixfold (i.e., by *f*_1_, *f*_2_, *f*_3_, *f*_5_, *f*_9_, and *f*_10_), DE twice (i.e., by *f*_4_ and *f*_7_), while the other algorithms once (i.e., SABA by *f*_6_ and ABC by *f*_8_). BA and FA did not outperform any other algorithm in the test.

In order to evaluate the quality of the results statistically, Friedman tests [29, 30] were conducted to compare the average ranks of the compared algorithms. Thus, a null hypothesis is placed that states the following: two algorithms are equivalent and therefore their ranks should be equal. When the null hypothesis is rejected, the Bonferroni-Dunn test [31] is performed. In this test, the critical difference is calculated between the average ranks of those two algorithms. If the statistical difference is higher than the critical difference, the algorithms are significantly different.

**Figure 2:**
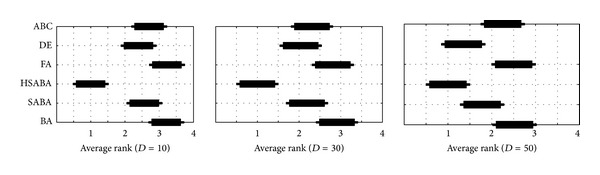
Results of the Friedman nonparametric test on the specific large-scale graph instances.

Three Friedman tests (Figure 2) were performed regarding data obtained by optimizing ten functions of three different dimensions according to five measures. As a result, each algorithm during the tests (also the classifier) was compared with regard to the 10 functions × 5 measures; this means 50 different variables. The tests were conducted at the significance level 0.05. The results of the Friedman nonparametric test can be seen in Figure 1 that is divided into three diagrams. Each diagram shows the ranks and confidence intervals (critical differences) for the algorithms under consideration with regard to the dimensions of the functions. Note that the significant difference between two algorithms is observed if their confidence intervals denoted by thickened lines in Figure 1 do not overlap.

The first two diagrams in Figure 1 show that the HSABA significantly outperforms the results of the other algorithms by solving the benchmark functions with dimensions *D* = 10 and *D* = 30. Although the other algorithms are not significantly different between each other the algorithms DE, SABA, and ABC are substantially better than the BA and FA. The third diagram shows that the HSABA remains significantly better than the ABC, FA, and BA, while the critical difference between DE and SABA was reduced and therefore becomes insignificant. On the other hand, this reduction caused the difference between DE and the other algorithms in the test, except SABA, to become significant.

## 5. Conclusion

The original BA algorithm gained better results by optimizing the lower-dimensional problems. When this algorithm was tackled with the harder problems of higher dimensions the results became poorer. In order to improve performance on high dimensional problems, almost two mechanisms are proposed in the publications: firstly, self-adaptation of control parameters and secondly hybridization of the original algorithm with problem-specific knowledge in the form of local search.

In this paper, both mechanisms were incorporated within the original BA algorithm in order to obtain the hybrid self-adaptive HSABA. Thus, the self-adaptive mechanism was borrowed from the self-adaptive jDE, while four different DE strategies were used as a local search. Finally, the evolution of the original BA via self-adaptive SABA and finally to hybrid HSABA is presented in detail.

On the one hand, the experimental work focuses on discovering the characteristics of the HSABA, while on the other hand, it focuses on the comparative study, in which the HSABA was compared with its predecessors BA and SABA as well as with other well-known algorithms, like FA, DE, and ABC. The results of this extensive work showed that the HSABA significantly outperformed the results of all the other algorithms in the tests. Therefore, the assumption taken from publications that self-adaptation and hybridization are appropriate mechanisms for improving the results of population-based algorithms was confirmed.

In the future work, this started work will be continued with experiments on the large-scale global optimization problems.

## Figures and Tables

**Algorithm 1:**
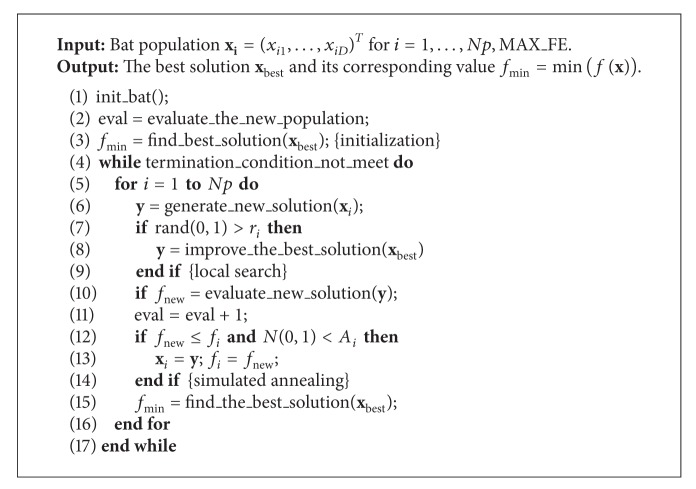
Pseudocode of the original bat algorithm.

**Algorithm 2:**
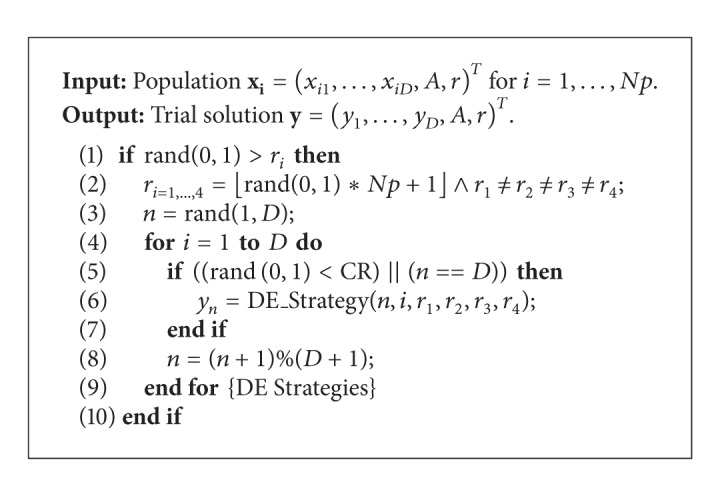
Modification in hybrid self-adaptive BA algorithm (HSABA).

**Algorithm 3:**
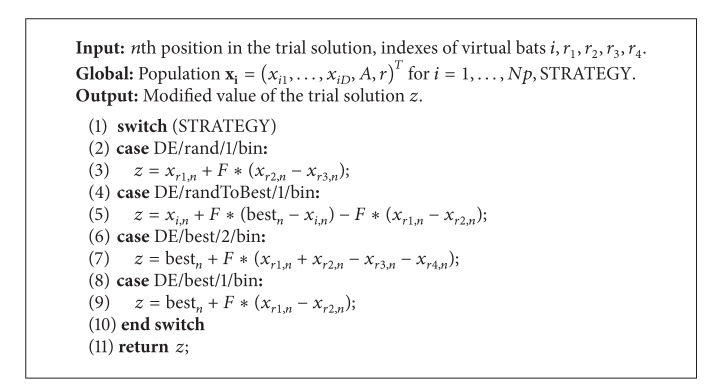
DE_Strategy function.

**Table 1:** Used DE strategies in the HSABA algorithm.

DE/rand/1/bin	*y*_*j*_ = *x*_*r*1,*j*_ + *F* · (*x*_*r*2,*j*_ − *x*_*r*3,*j*_)
DE/randToBest/1/bin	*y*_*j*_ = *x*_*i*,*j*_ + *F* · (best_*j*_ − *x*_*i*,*j*_) − *F* · (*x*_*r*1,*j*_ − *x*_*r*2,*j*_)
DE/best/2/bin	*y*_*j*_ = best_*j*_ + *F* · (*x*_*r*1,*j*_ + *x*_*r*2,*j*_ − *x*_*r*3,*j*_ − *x*_*r*4,*j*_)
DE/best/1/bin	*y*_*j*_ = best_*j*_ + *F* · (*x*_*r*1,*j*_ − *x*_*r*2,*j*_)

**Table 2:** Definitions of benchmark functions.

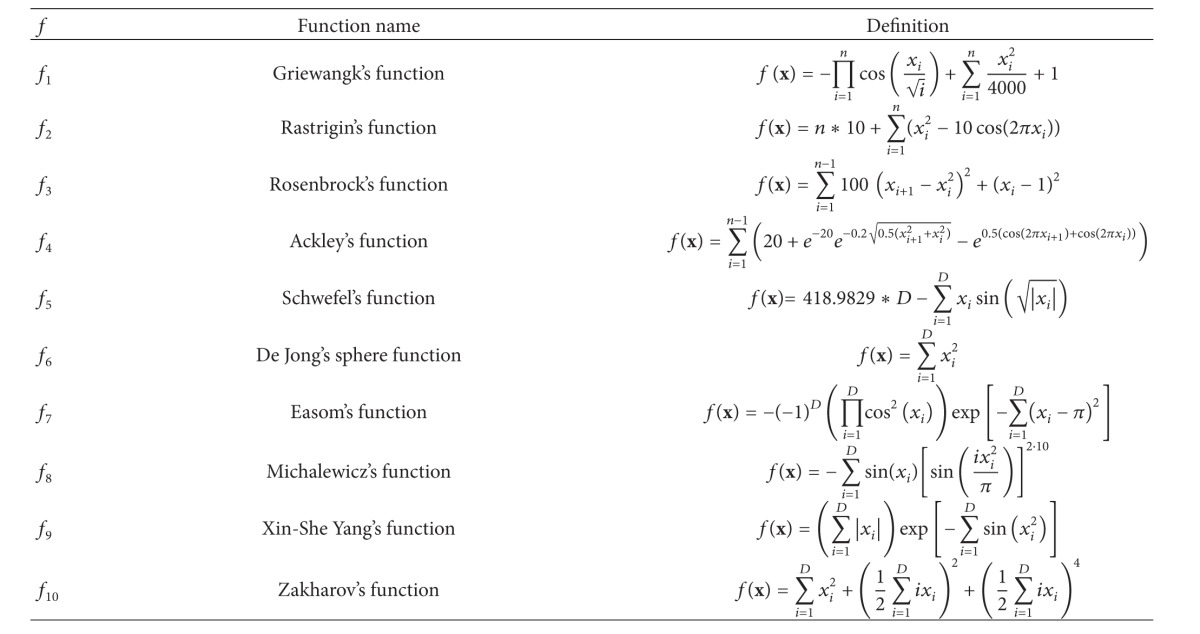

**Table 3:** Properties of benchmark functions.

*f*	f*	x*	Characteristics	Domain
*f*_1_	0.0000	(0,0,…, 0)	Highly multimodal	[−600,600]
*f*_2_	0.0000	(0,0,…, 0)	Highly multimodal	[−15,15]
*f*_3_	0.0000	(1,1,…, 1)	Several local optima	[−15,15]
*f*_4_	0.0000	(0,0,…, 0)	Highly multimodal	[−32.768,32.768]
*f*_5_	0.0000	(0,0,…, 0)	Highly multimodal	[−500,500]
*f*_6_	0.0000	(0,0,…, 0)	Unimodal, convex	[−600,600]
*f*_7_	−1.0000	(*π*, *π*,…, *π*)	Several local optima	[−2*π*, 2*π*]
*f*_8_	−1.8013^1^	(2.20319,1.57049)^1^	Several local optima	[0, *π*]
*f*_9_	0.0000	(0,0,…, 0)	Several local optima	[−2*π*, 2*π*]
*f*_10_	0.0000	(0,0,…, 0)	Unimodal	[−5,10]

^1^Valid for 2-dimensional parameter space.

**Table 4:** Detailed results (*D* = 10).

Evals.	Meas.	*f*_1_	*f*_2_	*f*_3_	*f*_4_	*f*_5_
4.00*E* + 02	Best	1.62*E* − 005	8.02*E* − 003	1.47*E* + 000	1.33*E* + 001	1.63*E* + 000
	Worst	1.03*E* + 00	3.14*E* + 02	3.58*E* + 005	2.00*E* + 001	1.42*E* + 003
	Mean	4.05*E* − 01	8.99*E* + 01	1.75*E* + 004	1.84*E* + 001	3.94*E* + 002
	StDev	2.27*E* − 01	5.87*E* + 01	7.10*E* + 001	2.00*E* + 001	1.68*E* + 002
	Mean	3.81*E* − 01	8.06*E* + 01	7.14*E* + 004	2.47*E* + 000	4.64*E* + 002

2.00*E* + 03	Best	7.90*E* − 06	6.77*E* − 03	2.32*E* − 002	8.39*E* − 003	4.56*E* − 003
	Worst	2.18*E* − 01	8.96*E* + 01	2.47*E* + 001	2.00*E* + 001	7.77*E* + 001
	Mean	2.91*E* − 02	1.54*E* + 01	5.00*E* + 000	1.01*E* + 001	1.52*E* + 001
	StDev	1.52*E* − 02	1.00*E* + 01	2.01*E* + 000	9.49*E* + 000	7.34*E* + 000
	Mean	5.36*E* − 02	2.19*E* + 01	6.12*E* + 000	7.09*E* + 000	1.79*E* + 001

1.00*E* + 04	Best	4.87*E* − 07	7.33*E* − 07	5.51*E* − 004	1.05*E* − 003	1.75*E* − 004
	Worst	1.19*E* − 01	2.22*E* + 01	8.82*E* + 000	2.00*E* + 001	1.65*E* + 001
	Mean	9.35*E* − 03	5.81*E* + 00	7.13*E* − 001	7.14*E* + 000	2.52*E* + 000
	StDev	1.88*E* − 04	9.71*E* + 00	8.03*E* − 003	6.56*E* + 000	5.65*E* − 002
	Mean	2.41*E* − 02	6.07*E* + 00	2.43*E* + 000	6.22*E* + 000	4.88*E* + 000

Evals.	Meas.	*f*_6_	*f*_7_	*f*_8_	*f*_9_	*f*_10_

4.00*E* + 02	Best	7.07*E* − 05	0.00*E* + 00	− 4.84*E* + 000	5.67*E* − 04	6.26*E* − 02
	Worst	3.28*E* + 00	0.00*E* + 00	− 2.14*E* + 000	3.37*E* − 03	9.55*E* + 01
	Mean	6.98*E* − 01	0.00*E* + 00	− 3.18*E* + 000	9.45*E* − 04	1.45*E* + 01
	StDev	2.62*E* − 01	0.00*E* + 00	− 3.02*E* + 000	6.70*E* − 04	2.20*E* + 00
	Mean	9.46*E* − 01	0.00*E* + 00	7.59*E* − 001	7.50*E* − 04	2.35*E* + 01

2.00*E* + 03	Best	8.13*E* − 06	0.00*E* + 00	− 6.51*E* + 000	5.66*E* − 04	6.18*E* − 05
	Worst	4.49*E* − 02	0.00*E* + 00	− 3.46*E* + 000	6.20*E* − 04	4.33*E* + 00
	Mean	4.75*E* − 03	0.00*E* + 00	− 4.70*E* + 000	5.74*E* − 04	3.52*E* − 01
	StDev	2.18*E* − 03	0.00*E* + 00	− 4.66*E* + 000	5.68*E* − 04	8.12*E* − 02
	Mean	9.10*E* − 03	0.00*E* + 00	8.55*E* − 001	1.32*E* − 05	8.74*E* − 01

1.00*E* + 04	Best	1.01*E* − 09	0.00*E* + 00	− 7.48*E* + 000	5.66*E* − 04	2.31*E* − 06
	Worst	6.85*E* − 03	0.00*E* + 00	− 3.92*E* + 000	5.71*E* − 04	7.67*E* − 01
	Mean	8.92*E* − 04	0.00*E* + 00	− 5.39*E* + 000	5.67*E* − 04	8.35*E* − 02
	StDev	8.47*E* − 06	0.00*E* + 00	− 5.19*E* + 000	5.66*E* − 04	2.79*E* − 02
	Mean	1.76*E* − 03	0.00*E* + 00	9.49*E* − 001	1.29*E* − 06	1.84*E* − 01

**Table 5:** Results according to dimensions.

*D*	Meas.	*f*_1_	*f*_2_	*f*_3_	*f*_4_	*f*_5_
10	Best	4.87*E* − 07	7.33*E* − 07	5.51*E* − 04	1.05*E* − 03	1.75*E* − 04
	Worst	1.19*E* − 01	2.22*E* + 01	8.82*E* + 00	2.00*E* + 01	1.65*E* + 01
	Mean	9.35*E* − 03	5.81*E* + 00	7.13*E* − 01	7.14*E* + 00	2.52*E* + 00
	StDev	1.88*E* − 04	9.71*E* + 00	8.03*E* − 03	6.56*E* + 00	5.65*E* − 02
	Mean	2.41*E* − 02	6.07*E* + 00	2.43*E* + 00	6.22*E* + 00	4.88*E* + 00

30	Best	6.68*E* − 05	8.48*E* − 04	1.19*E* − 03	1.73*E* − 01	6.94*E* − 03
	Worst	5.58*E* − 01	2.69*E* + 02	1.57*E* + 03	2.00*E* + 01	3.57*E* + 03
	Mean	7.71*E* − 02	4.63*E* + 01	1.02*E* + 02	9.44*E* + 00	2.70*E* + 02
	StDev	2.85*E* − 02	2.99*E* + 01	1.41*E* + 01	6.62*E* + 00	3.06*E* + 01
	Median	1.26*E* − 01	7.42*E* + 01	3.18*E* + 02	6.63*E* + 00	7.85*E* + 02

50	Best	7.16*E* − 05	2.03*E* − 02	5.77*E* − 05	1.71*E* − 03	3.06*E* − 02
	Worst	8.81*E* − 01	4.48*E* + 02	8.08*E* + 02	2.00*E* + 01	7.47*E* + 03
	Mean	1.70*E* − 01	9.88*E* + 01	7.07*E* + 01	1.11*E* + 01	9.00*E* + 02
	StDev	8.47*E* − 02	5.02*E* + 01	4.45*E* + 00	1.20*E* + 01	1.26*E* + 02
	Mean	2.29*E* − 01	1.27*E* + 02	1.89*E* + 02	8.59*E* + 00	2.12*E* + 03

Evals.	Meas.	*f*_6_	*f*_7_	*f*_8_	*f*_9_	*f*_10_

10	Best	1.01*E* − 09	0.00*E* + 00	− 7.48*E* + 00	5.66*E* − 04	2.31*E* − 06
	Worst	6.85*E* − 03	0.00*E* + 00	− 3.92*E* + 00	5.71*E* − 04	7.67*E* − 01
	Mean	8.92*E* − 04	0.00*E* + 00	− 5.39*E* + 00	5.67*E* − 04	8.35*E* − 02
	StDev	8.47*E* − 06	0.00*E* + 00	− 5.19*E* + 00	5.66*E* − 04	2.79*E* − 02
	Mean	1.76*E* − 03	0.00*E* + 00	9.49*E* − 01	1.29*E* − 06	1.84*E* − 01

30	Best	1.96*E* − 08	0.00*E* + 00	− 1.76*E* + 01	3.51*E* − 12	8.10*E* − 04
	Worst	2.17*E* − 01	0.00*E* + 00	− 7.09*E* + 00	2.02*E* − 11	1.22*E* + 02
	Mean	2.63*E* − 02	0.00*E* + 00	− 1.30*E* + 01	6.06*E* − 12	2.72*E* + 01
	StDev	1.29*E* − 03	0.00*E* + 00	− 1.36*E* + 01	3.85*E* − 12	1.37*E* + 00
	Mean	4.82*E* − 02	0.00*E* + 00	2.10*E* + 00	4.16*E* − 12	3.90*E* + 01

50	Best	4.72*E* − 05	0.00*E* + 00	− 2.83*E* + 01	1.21*E* − 20	4.57*E* − 01
	Worst	9.88*E* + 00	0.00*E* + 00	− 1.40*E* + 01	9.57*E* − 20	7.43*E* + 02
	Mean	5.59*E* − 01	0.00*E* + 00	− 1.95*E* + 01	2.44*E* − 20	2.34*E* + 02
	StDev	1.24*E* − 02	0.00*E* + 00	− 1.91*E* + 01	1.63*E* − 20	2.35*E* + 02
	Mean	1.97*E* + 00	0.00*E* + 00	3.25*E* + 00	1.99*E* − 20	2.04*E* + 02

**Table 6:** Obtained results of algorithms (*D* = 30).

*F*	Meas.	BA	SABA	HSABA	FA	DE	ABC
*f*_1_	Mean	1.16*E* + 00	1.03*E* + 00	7.71**E** − 02	6.65*E* − 01	1.05*E* + 00	1.09*E* + 00
	StDev	1.15*E* + 00	1.04*E* + 00	2.85*E* − 02	6.40*E* − 01	2.22*E* − 02	1.23*E* − 01

*f*_2_	Mean	9.28*E* + 02	7.02*E* + 02	4.63**E** + 01	2.44*E* + 02	2.28*E* + 02	7.33*E* + 01
	StDev	8.90*E* + 02	6.80*E* + 02	2.99*E* + 01	2.35*E* + 02	1.33*E* + 01	2.24*E* + 01

*f*_3_	Mean	2.84*E* + 06	3.67*E* + 05	1.02**E** + 02	1.12*E* + 02	4.57*E* + 02	5.18*E* + 02
	StDev	2.95*E* + 06	2.61*E* + 05	1.41*E* + 01	1.01*E* + 02	2.27*E* + 02	4.72*E* + 02

*f*_4_	Mean	2.00*E* + 01	2.00*E* + 01	9.44*E* + 00	2.11*E* + 01	1.77**E** + 00	7.17*E* + 00
	StDev	2.00*E* + 01	2.00*E* + 01	6.62*E* + 00	2.11*E* + 01	3.17*E* − 01	1.03*E* + 00

*f*_5_	Mean	9.45*E* + 03	9.13*E* + 03	2.70**E** + 02	6.78*E* + 03	7.57*E* + 03	2.64*E* + 03
	StDev	9.52*E* + 03	9.14*E* + 03	3.06*E* + 01	6.75*E* + 03	4.40*E* + 02	3.30*E* + 02

*f*_6_	Mean	5.87*E* − 02	1.45**E** − 05	2.63*E* − 02	5.19*E* + 00	1.77*E* + 02	1.63*E* + 02
	StDev	6.53*E* − 05	1.46*E* − 05	1.29*E* − 03	5.14*E* + 00	7.12*E* + 01	1.96*E* + 02

*f*_7_	Mean	0.00*E* + 00	0.00*E* + 00	0.00*E* + 00	− 3.81*E* − 30	− 2.76**E** − 175	− 1.76*E* − 136
	StDev	0.00*E* + 00	0.00*E* + 00	0.00*E* + 00	− 3.73*E* − 30	0.00*E* + 00	8.79*E* − 136

*f*_8_	Mean	− 8.62*E* + 00	− 8.51*E* + 00	− 1.30*E* + 01	− 5.15*E* + 00	− 1.07*E* + 01	− 2.30**E** + 01
	StDev	− 8.39*E* + 00	− 8.36*E* + 00	− 1.36*E* + 01	− 5.35*E* + 00	6.70*E* − 01	6.98*E* − 01

*f*_9_	Mean	1.57*E* − 11	1.41*E* − 11	6.06**E** − 12	1.70*E* − 04	2.46*E* − 11	1.10*E* − 11
	StDev	1.03*E* − 11	1.08*E* − 11	3.85*E* − 12	4.72*E* − 05	1.20*E* − 12	1.91*E* − 12

*f*_10_	Mean	2.76*E* + 02	2.04*E* + 02	2.72**E** + 01	1.32*E* + 04	3.78*E* + 01	2.53*E* + 02
	StDev	2.82*E* + 02	2.17*E* + 02	1.37*E* + 00	1.32*E* + 04	8.74*E* + 00	3.15*E* + 01
